# Supplementary material for: Association between Patients’ Body Mass Index and the Effect of Monophasic Pulsed Microcurrent Stimulation on Pressure Injury Healing
Source: Biomedicines. 2023 Aug 25;11(9):2379. doi: 10.3390/biomedicines11092379 (PMC10526075; doi:10.3390/biomedicines11092379)
Supplement: Supplementary file 1 [file biomedicines-11-02379-s001.zip › biomedicines-2504556-SI.pdf]

Supplementary material Table S1: Information of patients participated in the study.

Patient	Age (years)	Sex	BMI	Albumin (g/dL)	WBCs (μL)	Hb	CRP	Nutrition	Underlying disease	Location of pressure ulcers	Duration of illness	Total DESIGN-R score
A	80	Female	15.4	3.3	5.1	12	0.27	Oral ingestion	Lumbar compression fracture	Sacrum	12 months	19 (D3-e3s3i0g3n0P12)
B	90	Female	16.8	3.1	10.3	10.5	6.33	Tube feeding	Cerebral infarction	Sacrum	12 months	25 (D3-e3s9i1g3n0P9)
C	80	Female	19.3	2.5	9.4	10.6	0.54	Tube feeding	Parkinson’s disease	Sacrum	10 months	26 (D3-e3s6i1G4n0P12)
D	62	Female	19.4	3.8	7.6	14.2	0.02	Oral ingestion	Diabetes	Sacrum	≥2 months	18 (D3-e3s3i0g3n0P9)
E	92	Female	16.9	2.6	7.2	11.1	0.27	Tube feeding	Pyelonephritis	Sacrum	≥5 months	18 (D3-e3s3i0g3n0P9)
F	84	Female	18.7	2.3	8.5	10.4	2.01	Tube feeding	Subarachnoid hemorrhage	Sacrum	10 months	23 (D3-e3s3i0G5N3P9)
G	89	Female	18.8	3.1	5.5	10.5	0.16	Oral ingestion	Total knee arthroplasty	Thoracic spine	≥12 months	20 (D3-e3s3i1G4n0P9)
H	80	Male	16.8	2.7	5.7	13.3	1.35	Tube feeding	Normal pressure hydrocephalus	Coccyx	20 months	15 (D3-e3s3i0g3n0P6)
I	85	Male	18.2	2	7.4	8.4	4.92	Tube feeding	Cerebral hemorrhage	Left ilium	≥8 months	19 (D3-e3s3i0G4N3P6)
J	85	Male	18.5	3	10.7	7.1	6.76	Oral ingestion	Metastatic spinal cord tumor	Right greater trochanter	≥4 months	25 (D3-e3s6i0G4N3P9)
K	82	Male	18.9	2.7	9.4	9.2	4.38	Tube feeding	Hypoxic encephalopathy	Right fibula	3 months	18 (D3-e3s6i0g3n0P6)

Supplementary material Figure S1: Figures of wounds.

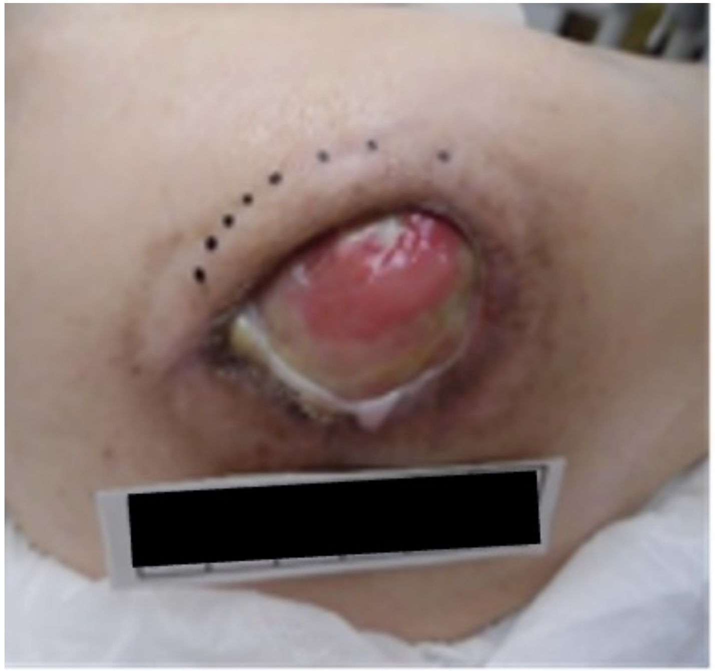

Before microcurrent treatment

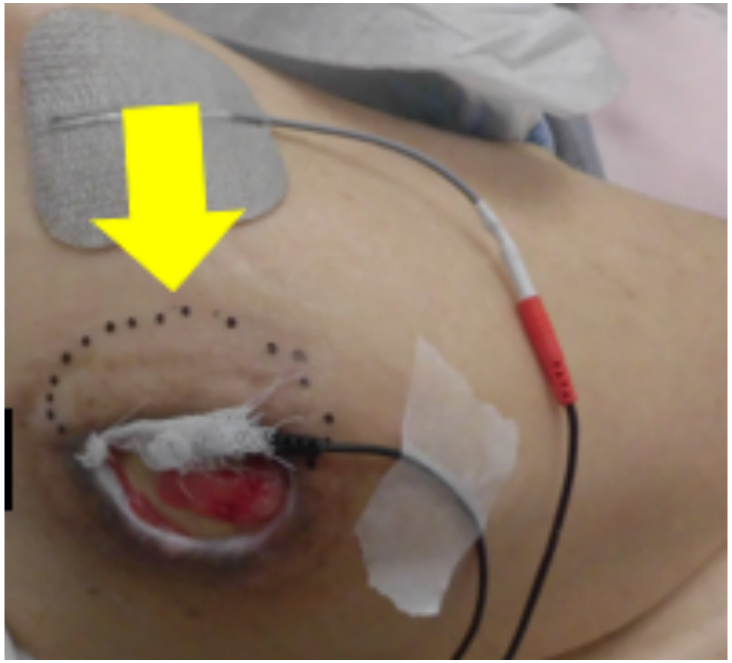

After microcurrent treatment
